# Thermal Characterisation of Hybrid, Flip-Chip InP-Si DFB Lasers

**DOI:** 10.3390/mi14020381

**Published:** 2023-02-03

**Authors:** David Coenen, Huseyin Sar, Herman Oprins, Aleksandrs Marinins, Yannick De Koninck, Stuart Smyth, Yoojin Ban, Joris Van Campenhout, Ingrid De Wolf

**Affiliations:** 1 Department of Materials Engineering, KU Leuven, 3000 Leuven, Belgium; 2 Imec, 3001 Leuven, Belgium; 3 Sivers Photonics, Glasgow G72 0BN, UK

**Keywords:** hybrid laser integration, thermal modelling, thermal characterisation, silicon photonics

## Abstract

WA detailed thermal analysis of a hybrid, flip-chip InP-Si DFB laser is presented in this work. The lasers were experimentally tested at different operating temperatures, which allowed for deriving their thermal performance characteristics: the temperature dependence of threshold current, lasing slope, and output spectrum. Using these data, the laser thermal resistance was calculated (*R*_th_ = 75.9 K/W), which allows for predicting the laser temperature during operation. This metric is also used to validate the thermal finite element models of the laser. A sensitivity study of the laser temperature was performed using these models, and multiple routes for minimising both the laser thermal resistance and thermal coupling to the carrier die are presented. The most effective way of decreasing the laser temperature is the direct attachment of a heat sink on the laser top surface.

## 1. Introduction

The exponential increase in data centre traffic has driven the development of high-bandwidth, high-efficiency, and low-latency interconnects. One very promising technology for reaching these demands consists of copackaged silicon photonics (SiPho) transceivers [1]. Close integration to a network switch minimises interconnect losses, but introduces additional thermal challenges such as heat flux coming from electronics and difficulties in removing heat from the transceiver itself. One possible route for increasing optical I/O bandwidth is by scaling the number of wavelength division multiplexing (WDM) channels [2]. While keeping the bitrate per channel constant, the capacity of the optic fibres is increased by transmitting multiple channels in parallel. Scaling the number of WDM channels requires appropriate multiple-wavelength light sources. Because of the indirect band gap of Si, it is unsuitable for on-chip light generation. Recently, multiple approaches have been considered for integrating light sources within the SiPho transceiver. Monolithic integration consists of the direct epitaxial growth of III–V material for optical gain on the SiPho wafer. This method uses nanoridge engineering for the trapping of defects generated by the lattice mismatch between the materials [3]. The second approach consists of integrating a separately manufactured laser die into the SiPho platform. This can be done by microtransfer printing [4] or flip-chip bonding [5]. Alternatively, heterogeneous die-to-wafer bonding [6] is also possible. The focus of this work is hybrid integration by means of the flip-chip bonding of a InP DFB laser to a SiPho carrier die and the thermal characterisation of the bonded laser.

The laser temperature during operation can be measured indirectly by measuring the wavelength shift of the output spectrum [7] or directly with IR cameras [8,9,10]. The latter method is less suited for flip-chipped lasers because of the difficulty of accessing the bottom side of the laser, so the indirect method using the laser spectrum is used. The laser generates heat during operation, and if there are no low-thermal-resistance paths to a heat sink, the laser temperature increases significantly. This negatively impacts its performance and reliability. Reliability is not studied here, but the thermally dependent laser performance is characterised and modelled. In the literature, multiple methods for the thermal management of lasers for Si photonics have been proposed. First, the buried oxide (BOX) layer can be locally removed for improved heat removal [11]. Alternatively, if this layer cannot be removed, a thermal shunt with a highly thermally conductive material can be produced [12]. Second, thermal management on the package level can be performed with the addition of a heat spreader either connected to a thermoelectric cooler element [13] or directly connected to a forced air convection heat sink [14]. For highly efficient and reliable operation, it is imperative that the laser temperature is minimised by means of a thermally aware design.

After the device presentation in Section 2, the experimental thermal characterisation of the laser is discussed in Section 3. The focus is on the laser L–I–V and spectrum, and the measurement results were used for the calculation of the laser thermal resistance. The obtained value is used for model validation in Section 4; after that, the thermal model is used to explore an improved thermal design.

## 2. Devices and Experiments

**Figure 1:**
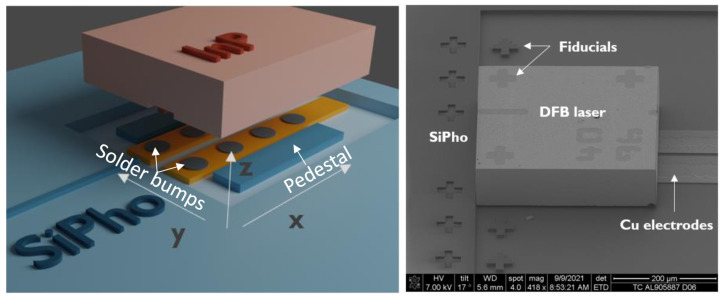
Hybrid integration of InP laser by flip-chip bonding on SiPho die. Pedestals are present for mechanical support. Figures were adapted from [15].

Figure 1 (left) shows a sketch of the InP laser and the landing zone on the SiPho die. There are two pedestals on the side that provide mechanical support to the laser and the accurate z-alignment of the laser with respect to the SiPho die. In the centre, there are two Cu electrodes with solder bumps that are used for the electrical connection of the laser, which has coplanar anode and cathode contacts, eliminating the need for a wire bond. Optical coupling to the SiPho die is performed by emitting light from one facet of the laser, which propagates into a tapered SiN waveguide. With an inplane laser placement accuracy of 254 nm (3σ) and 284 nm (3σ) for x and y, respectively, a coupling loss of 1.5 ± 0.5 dB was obtained [15]. The facet on the other side of the laser had a highly reflective coating, creating a mirror for the laser cavity. Figure 1 (right) shows a microscopic image of the laser: the laser dimensions were 350 × 300 × 100 μm${}^{3}$.

Five different laser dies were measured to provide statistics. For each laser, the measurements consisted of two parts:

1. Measurement of the light amplitude and laser voltage by sweeping the input current between 0 and 100 mA, resulting in light–current–voltage (LIV) curves.
2. Measurement of the optical spectrum at six different temperatures in the range of 25–75 °C with increments of 10 °C, and for four different currents in the range of 15–60 mA with increments of 15 mA, for each temperature.

The laser temperature was controlled by placing the SiPho die on a heater chuck. The input current was controlled by probing the Cu electrodes, which extended below the laser (Figure 1). After coupling into the SiN waveguide, the light was coupled into an optical fibre through a grating coupler and processed with an optical-spectrum analyser (OSA).

The thermal resistance is the most important thermal characteristic of the laser. Here, it is defined as the volumetric average laser mesa temperature increase per unit of dissipated power, expressed in units of K/W. It is difficult to directly measure the laser temperature due to the small scale and inaccessible location of the laser mesa. Because of this, the laser temperature (and thermal resistance) is measured indirectly by using the spectral data at different temperatures and currents. By determining the shift of the main emission wavelength with respect to the temperature and thermal power, the thermal resistance can be calculated [7,12,16]:

(1) $$R_{\mathrm{th}}={\left(\frac{\partial \lambda }{\partial T}\right)}^{-1}\left(\frac{\partial \lambda }{\partial P}\right)$$

## 3. Experimental Resultss

### 3.1. Laser L-I-V

**Figure 2:**
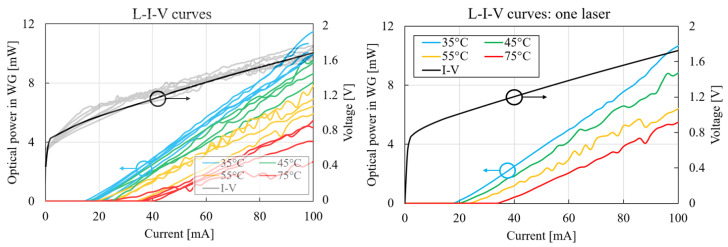
Laser L–I–V characteristics with increasing temperature as parameter (T=35 °C:75 °C). The I–V curve was taken at T=25 °C and showed no significant temperature dependence.

**Figure 3:**
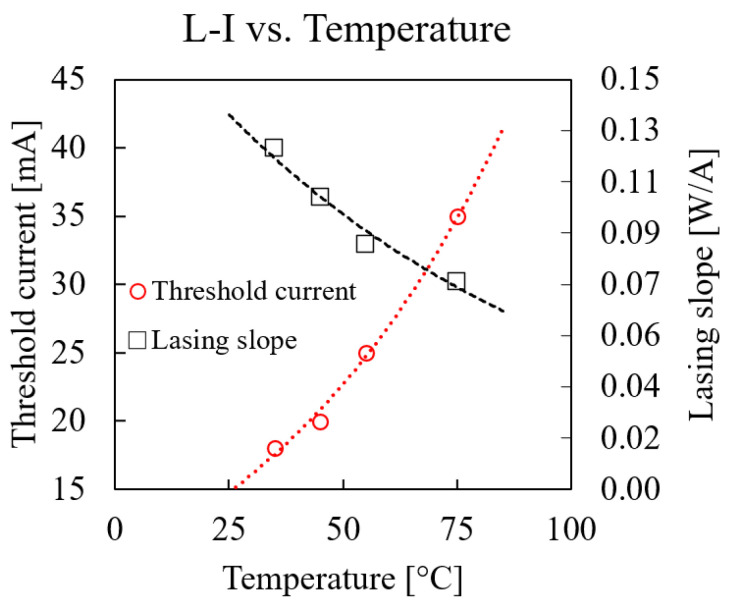
Laser L–I temperature dependence: lasing slope (black) and threshold current (red).

L–I–V measurement results are shown in Figure 2: the curves were obtained for chuck temperatures between 35 and 75 °C. The optical power was waveguide-coupled: the grating coupler losses were subtracted from the measured optical power, and the coupling loss from laser to waveguide was included. There were two main temperature-driven effects: the threshold current, which is the current at which the laser starts emitting light and increases, and the lasing slope decreases. The values of both these effects are plotted in Figure 3 for a specific laser die as a function of chuck temperature. Both could be described by an exponential function, as shown by the fit to the measurement data. The underlying effect that causes the shift in threshold current and the decrease in lasing slope is the lower carrier lifetime due to an increased recombination rate. The main nonradiative recombination mechanism in this type of laser is Auger recombination, which is temperature-sensitive [17,18]. The fitting parameters for these functions are useful for building a compact thermal laser model, which is demonstrated in the next section.

**Figure 4:**
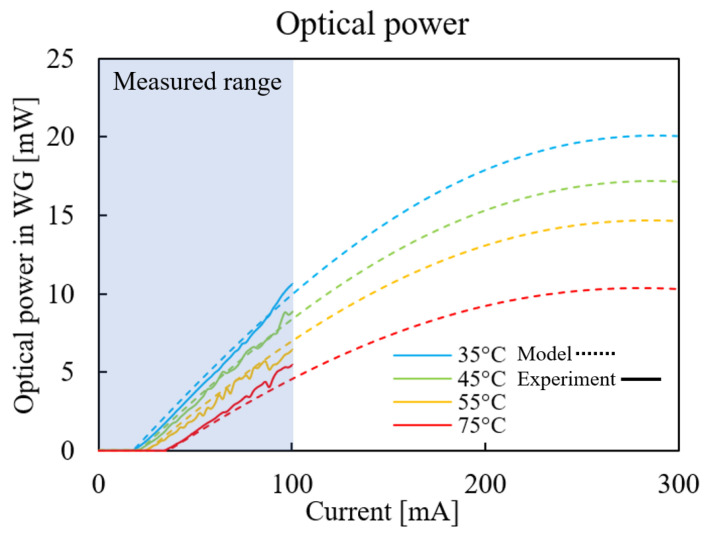
Laser L–I characteristic and model fit.

The laser threshold current shift is described with the following equation [7]:

(2) $$I_{\mathrm{th}}=I_{0}e^{T/T_{0}}$$

where $T_{0}$ is the laser-characteristic temperature, and $I_{0}$ is a fitting parameter. The lasing slope (W/A) was calculated according to

(3) $$\eta_{\mathrm{slope}}=\eta_{0}e^{-T/T_{1}}$$

where $\eta_{0}$ is a fitting parameter. The extracted laser fitting parameters and characteristic temperatures could lastly be combined into a compact laser model for predicting output optical power, taking into account performance degradation due to self-heating [1,7].

(4) $$P_{\mathrm{opt}}=\eta_{0}e^{\frac{-(R_{\mathrm{th}}\left(P_{\mathrm{el}}-P_{\mathrm{opt}}\right)+T_{a})}{T_{1}}}\left(I-I_{0}e^{\frac{R_{\mathrm{th}}\left(P_{\mathrm{el}}-P_{\mathrm{opt}}\right)+T_{a}}{T_{0}}}\right)$$

where $R_{\mathrm{th}}$ is the thermal resistance, $P_{\mathrm{el}}$ and $P_{\mathrm{opt}}$ are the electrical and optical power, respectively, and $T_{a}=25$ °C is the ambient temperature. The extraction of the laser thermal resistance from the measurement data is elaborated in Section 3.3. Equation 4 is implicit as $P_{\mathrm{opt}}$ is on the left- and right-hand sides, and was solved iteratively; the result is shown in Figure 4. The measurements were conducted for a maximal pumping current of 100 mA, and the model allows for extrapolating to higher current levels. Thermal rollover was identified at these higher current levels: at this point, an increase in current results in a decrease in output power. The fitting parameters used for the compact model are summarised in Table 1.

### 3.2. Laser Spectrum

**Figure 5:**
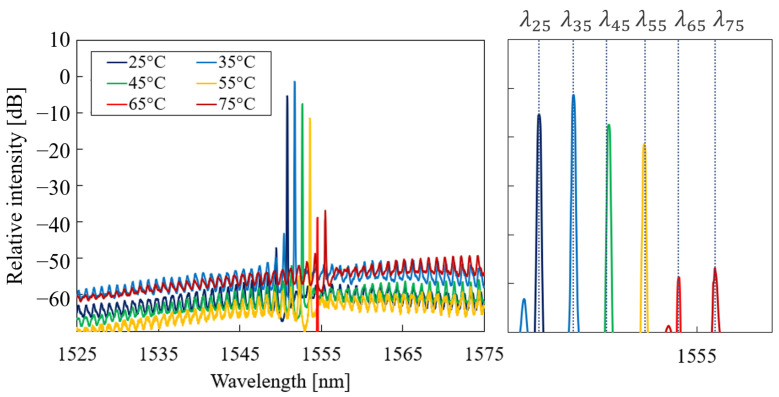
Measured laser spectrum for different temperatures. The detailed view shows the main extracted emission wavelength. The intensity of power spectral density was measured at a resolution of 20 pm.

**Figure 6:**
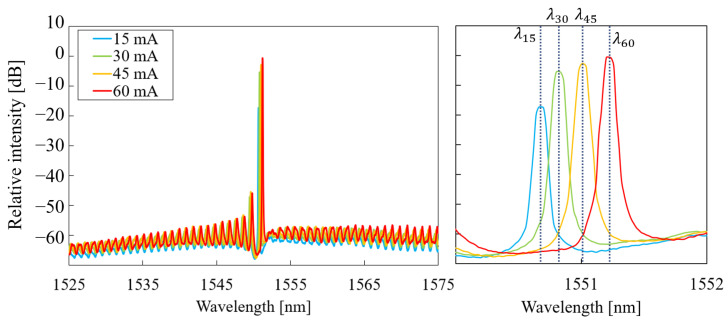
Measured laser spectrum for currents. The detailed view shows the main extracted emission wavelength. The intensity of power spectral density was measured at a resolution of 20 pm.

In this section, the measurement of the laser spectrum is discussed. The spectrum was measured for temperatures in the range of T=25–75 °C and at each temperature for currents in the range of 15–60 mA. Figure 5 shows the laser spectrum for a constant current (I=30 mA) and different chuck temperatures. The magnification on the main emission wavelength shows a red shift in the peak in optical power for increasing temperatures. This behaviour was expected for this laser type, as the gain spectrum in the AlGaInAs MQW active region shifts to higher wavelengths at increased temperatures levels [17]. The amplitude of the emission peaks in Figure 5 did not follow the expected exponential decrease with respect to temperature. This was caused by variable grating coupler losses at different temperatures because of fibre alignment inaccuracy. The measurement results are plotted for constant chuck temperature and variable current in Figure 6. Similarly to the results in Figure 5, the main emission wavelength showed a red shift via increasing the pumping current.

**Figure 7:**
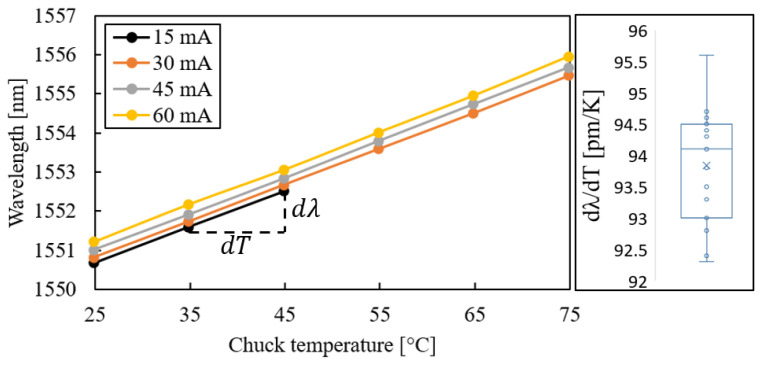
Laser wavelength as a function of chuck temperature for one laser and the box plot of linear fit through all slopes of the five characterised lasers.

As the next step, the laser-characteristic slopes from Equation (1) were extracted from the measurement results. The wavelength shift with respect to the chuck temperature is shown in Figure 7; the value ∂λ/∂T=94.1±1.5 pm/K was obtained.

The calculation of the second laser-characteristic slope ∂λ/∂P was more complicated and required the determination of the thermal power dissipation inside the laser for each datum.Using the definition of wall plug efficiency $WPE=P_{\mathrm{opt}}/P_{\mathrm{el}}$, combined with the steady-state power balance $P_{\mathrm{el}}=P_{\mathrm{opt}}+P_{\mathrm{th}}$, thermal power $P_{\mathrm{th}}$ is:

(5) $$P_{\mathrm{th}}=V\left(I\right)\cdot I\cdot \left(1-WPE\left(I,T\right)\right)$$

WPE is typically a function of operating temperature and pumping current; in order to derive an expression in the function of known parameters, optical and electrical power are expressed as follows:

(6) $$P_{\mathrm{opt}}=\eta_{\mathrm{slope}}\left(I-I_{\mathrm{th}}\right)$$

(7) $$P_{\mathrm{el}}=V_{\mathrm{op}}I+R_{s}I^{2}$$

where $V_{\mathrm{op}}$ is the operating forward diode bias at which conduction starts, and $R_{s}$ is the series resistance found as the slope of the I–V curve in the conduction regime. In this analysis, both $V_{\mathrm{op}}$ and $R_{s}$ were assumed to be constant with respect to temperature, which was confirmed within the measured range (see Figure 2). Equation (6) is a more compact version of Equation (4). By combining Equations (3), (6), and (7), we obtained:

(8) $$WPE\left(I,T\right)=\frac{\eta_{0}e^{-T/T_{1}}\left(I-I_{0}e^{T/T_{0}}\right)}{I(V_{\mathrm{op}}+R_{s}I)}$$

**Figure 8:**
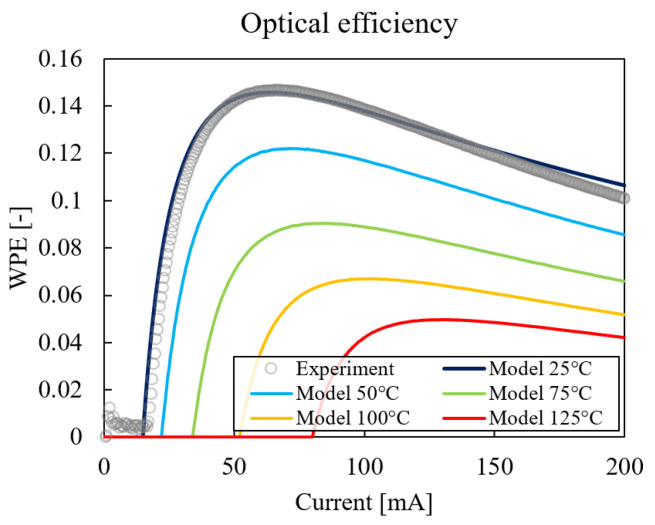
Measured wall plug efficiency as a function of current (gray circle) and model fit (black). Model extrapolation up to T=125 °C.

Using Equation (8), the wall plug efficiency was calculated at room temperature (T=25 °C) and for a current up to 200 mA; the result was compared with the experimental measurement of WPE (bar test data, no coupling losses included) as shown in Figure 8. A good match was obtained between the model and the experiment.

**Figure 9:**
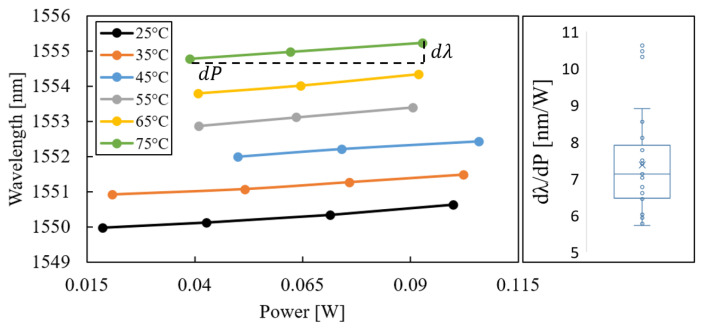
Laser wavelength as a function of thermal power and box plot of linear fit through all measured slopes of the five characterised lasers.

Using the model for wall plug efficiency, the WPE of all possible combinations of operating temperatures and currents was calculated. The results are shown in Figure 8 for temperatures in the range of 25–125 °C. Lastly, these results were used in conjunction with Equation (5) for the calculation of the thermal power dissipation at each data point (see Figure 6). The resulting laser-characteristic slopes were plotted in Figure 9, and a value of ∂λ/∂P=7.12±1.40 nm/W was obtained.

### 3.3. Thermal Resistance

**Figure 10:**
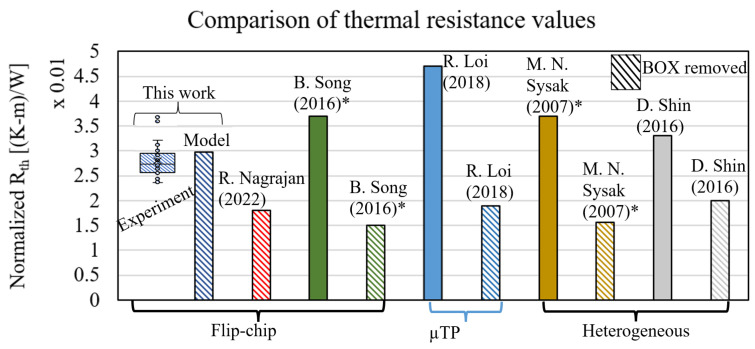
Thermal resistance from experiments, model, and values from literature. R. Nagarajan (2022) [21] and B. Song (2016) [20] report results on flip-chip integrated lasers. R. Loi (2018) [11] reports µprinted lasers and M.N. Sysak (2007) [7] and D. Shin (2016) [19] report on heterogeneous laser integration. The thermal resistance was normalised as the laser length × thermal resistance product. Results indicated with ${}^{*}$ are simulation results.

Both laser-characteristic slopes were determined, and these results were combined in order to calculate the laser thermal resistance (Equation (1)). A value of $R_{\mathrm{th}}=75.9\pm 16.3$ K/W was obtained. The main contribution to the measurement uncertainty stemmed from ∂λ/∂P, for which the measured current and voltage were used (laser I–V). The obtained value for thermal resistance could be compared to other work by normalising the result with respect to laser length. The laser-length and thermal-resistance product of this work was 2.6 $\times \;10^{-2}$ (K-m)/W. In Figure 10, this value is compared with other reported values in the literature [7,11,19,20,21]. The normalised thermal resistance lay in the range of 1.5–4.7 $\times \;10^{-2}$ (K-m)/W, placing the laser studied in this work inside the expected range. The values indicated with ${}^{*}$ in Figure 10 are based on simulations and are not demonstrated with measurements. Lastly, the values indicated with full colors are for laser integration on SOI, while the striped results were obtained without a buried oxide (BOX) layer below the laser. The laser integration in this work was categorised as a flip-chip method without a BOX layer.

**Figure 11:**
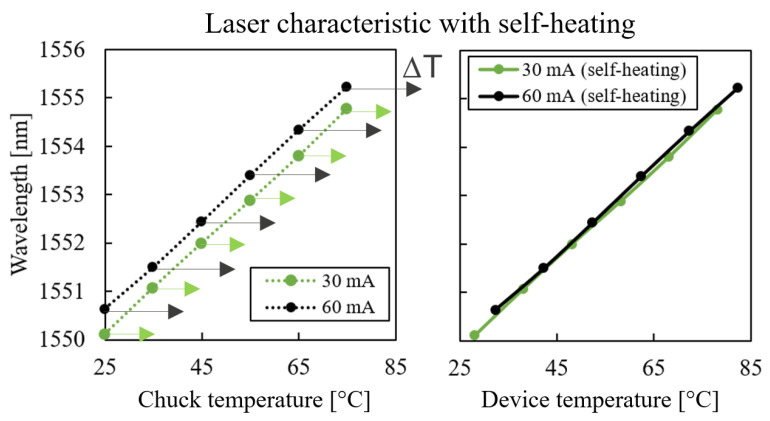
Laser wavelength as a function of (**left**) chuck temperature and (**right**) actual mesa temperature.

The laser-characteristic slopes (∂λ/∂T) in Figure 7 were measured for different current levels and plotted as a function of chuck temperature. However, as the laser thermal resistance was known, it was possible to calculate the actual laser temperature, which was higher than the chuck temperature due to self-heating. Using $T=T_{\mathrm{chuck}}+R_{\mathrm{th}}P_{\mathrm{th}}$, the self-heating temperature shift was calculated for two curves at constant current as shown in Figure 11. Measurement points at 30 mA were less affected by self-heating compared to points at 60 mA. If the measurement points were plotted as a function of actual laser temperature instead of chuck temperature, it would become clear that the curves coincide (Figure 11, right). This signifies that the wavelength shift with respect to temperature was current-independent, as expected. This has two implications: first, the extracted value of the thermal resistance $R_{\mathrm{th}}$ from the measurement data was accurate, as this value was used for the calculation of the shifted curves that coincided in Figure 11. If another value of $R_{\mathrm{th}}$ was used, the curves would not coincide. Second, in [12], the laser was used in pulsed mode for the determination of ∂λ/∂T in order to prevent self-heating. We show here that operating the laser in CW mode is also possible, as the self-heating simply shifts the curves, but does not change the slope itself.

## 4. Modelling Analysiss

**Figure 12:**
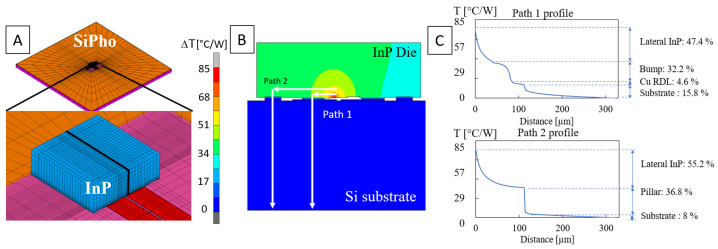
(**A**) Finite element model of SiPho die with the detail of the InP laser laser; (**B**) laser cross-section showing temperature contours; (**C**) temperature profiles through solder bump and pedestal.

Thermal finite element models of the laser were produced using MSC Marc [22]. The computational domain consisted of the SiPho die (6 × 6 mm${}^{2}$) and the laser (350 × 300 μm^2^) that was flip-chipped on top. Only heat conduction was calculated; the convective heat transfer on the top side of the dies was modelled with an equivalent heat transfer coefficient of natural convection. The SiPho die was assumed to have been placed on a temperature-controlled chuck, which was an isothermal surface in the simulation. The sidewalls of the SiPho die were adiabatic. The parasitic resistive losses of the current path from the Cu electrodes through the laser were not taken into account: all heat generation was assumed to have occurred inside the laser mesa, the active optical part. This heat generation was added uniformly across the laser mesa and normalised to 1 Watt. The computational mesh is shown in Figure 12, and it was sufficiently refined in order to obtain a mesh-size-independent solution. The material properties used for the simulation are summarised in Table 2.

### 4.1. Model Validation

The average laser mesa temperature was extracted from the thermal finite element simulation. The input thermal power was set to 1 Watt; thus, the resulting temperature was equal to the thermal resistance in units of K/W [23]. The overview of the model is shown in Figure 12A, and a cross-section of the simulated temperatures is plotted in Figure 12B. The modelled thermal resistance was $R_{\mathrm{th}}=85.7$ K/W, which matched the experimental result well ($R_{\mathrm{th}}=75.9\pm 16.3$ K/W) and lay within the measured range (Figure 10).

### 4.2. Improving the Laser Thermal Paths

#### 4.2.1. Temperature Profile

The last section deals with the analysis of the validated thermal finite element model. In Figure 12B, two paths are indicated from the heat source to the heat sink. Path 1 was formed by a conduction path through the solder bumps into the Si substrate. Path 2 was formed via conduction through the pedestals. Additionally, a small fraction of heat was lost ambiently by means of natural convection on the top side of the laser. However, this was a negligible amount (<0.04%) due to the small surface area of the laser. In Figure 12C, the temperature profile along both conduction paths is plotted. In both cases, approximately ∼50% of the temperature drop was attributed to thermal resistance inside the laser itself due to lateral conduction. About one-third of the temperature drop was in either the solder bump or the pedestal, and the remaining temperature drop occurred through the Si substrate. The relatively large temperature drop inside the laser itself signifies that this hybrid integration approach achieved good thermal performance. The main thermal resistance contribution came from inside the laser itself, and was not caused by the integration method and flip-chip process. In order to allow for the direct coupling of light from the laser into the SiN waveguide, a recess was etched into the SOI wafer. The depth was chosen such that the laser optical mode had the same z-coordinate as that of the SiN waveguide. The process of recess etching creates a plane for the laser to be bonded below the buried oxide layer (BOX). The BOX is typically the main contributor of the total thermal resistance for photonic devices in the SOI platform; however, in this case, it was removed for optical coupling purposes, also lowering thermal resistance.

#### 4.2.2. Heat Sink

**Figure 13:**
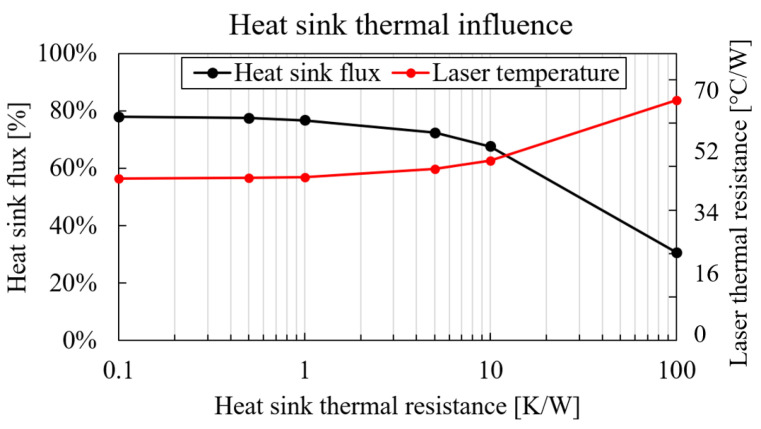
Laser thermal resistance (temperature, red) and heat sink flux (black) as a function of heat sink thermal resistance.

As the next step, the thermal boundary conditions were varied, and their effect on laser temperature was recorded. The isothermal bottom face of the SiPho die was replaced with equivalent thermal resistance of 4 K/W, representing packaging conditions (e.g., integration on PCB with BGA). The natural convection boundary condition on the top side of the laser was replaced with variable thermal resistance $R_{\mathrm{top}}=0.1$–100 K/W, representing the direct attachment of a heat sink [24]. The simulation result is shown in Figure 13; the heat sink flux (black) reaches almost 80% of the total heat for very efficient heat sinks, and the laser temperature (red) is reduced with −39%. However, the very small values for heat sink thermal resistance were not very realistic. Due to the laser size (350 × 300 μm${}^{2}$), there was a significant contribution of thermal spreading resistance inside the heat sink. Using data from commercially available heat sinks, a more realistic heat sink thermal resistance lies in the range of $R_{\mathrm{hs}}=40$–100 K/W. At a $R_{\mathrm{hs}}=100$ K/W, there was still a −20% reduction in laser temperature.

#### 4.2.3. Thermal Coupling

The next focal point in the thermal design was the heat spreading from the laser into the SiPho die. This heat spreading caused a thermal coupling between the laser and nearby photonic devices in the SiPho die. This thermal coupling is an unwanted effect that can detune thermally sensitive devices, such as ring-based resonators [25,26] or thermo-optical phase shifters. The thermal coupling was calculated by extracting a temperature profile inside the SiPho die from the simulation and normalising it with respect to the maximal temperature inside the laser:

(9) $$C_{\mathrm{thermal}}\left(x\right)=\frac{T(x)}{T_{\max}}\cdot 100\%$$

**Figure 14:**
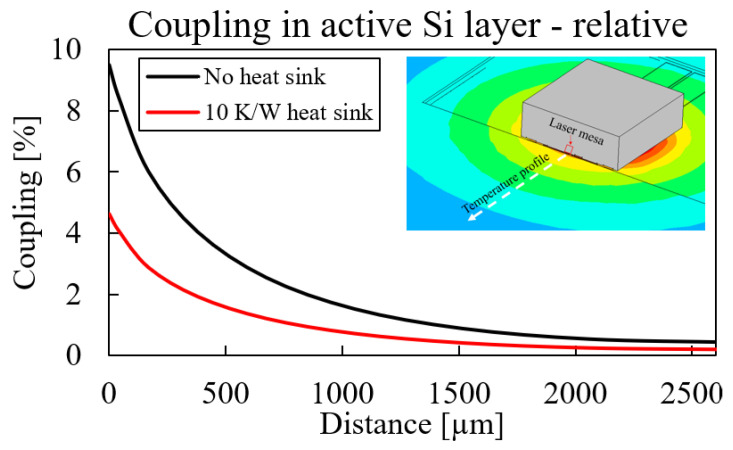
Thermal coupling to the SiPho die as a function of distance from the laser.

The thermal coupling for two different cases is shown in Figure 14: without a heat sink (black) and with a heat sink (red). The addition of a heat sink significantly decreased thermal coupling: a 10 K/W heat sink decreased the coupling by a factor of 2. This large impact is explained in Figure 13: about ∼70% of the total generated heat was removed through the top side of the laser with a 10 K/W heat sink, greatly decreasing the amount of heat that spread inside the SiPho die. Additionally, the absolute thermal coupling was decreased even more, as the laser temperature $T_{\max}$ was also lowered by the heat sink.

#### 4.2.4. Underfill

**Figure 15:**
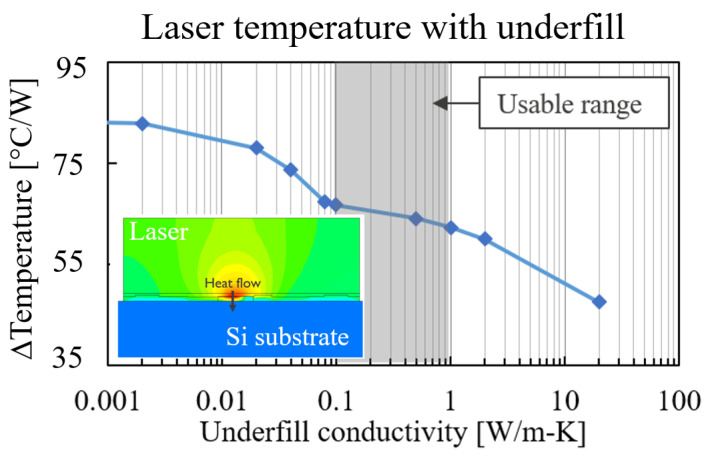
Laser temperature rise vs. underfill thermal conductivity. Insert shows the temperature contour plot and heat flux through the underfill into the Si substrate.

The final investigated aspect with the model was the addition of an epoxy underfill below the laser. Because there was a large fraction of thermal resistance attributed to heat spreading in the InP (Figure 12), there was potential for improving the thermal performance by creating a direct heat transfer path from the laser mesa into the Si substrate. This partially mitigated the heat spreading in the InP. The underfill was modelled by filling all empty space below the laser with a new material. The laser temperature as a function of underfill thermal conductivity is shown in Figure 15 for a wide range of values. For very low thermal conductivity, the laser temperature approached the limit case of no underfill. The expected range of thermal conductivity was 0.1–1 W/m-K, resulting in a temperature reduction of 23–31%. The insert in Figure 15 shows that the temperature in the laser was uniform because the underfill helped in heat spreading, and there was a temperature gradient in the underfill right below the laser mesa. This indicates that a part of the heat directly flowed into the Si substrate, effectively making the underfill a thermal shunt.

## 5. Conclusions

The hybrid integration of on-chip light sources for Si photonic applications was studied in this work; more specifically, the thermal performance of a flip-chip bonded III–V (InP) DFB laser was assessed. Through experimental analysis, the temperature dependence of the main laser characteristics was obtained: the laser threshold current, lasing slope, and output spectrum. The laser could be thermally characterised in both CW operation and low-duty-cycle pulsed operation. The induced self-heating during CW caused a shift of the laser curves ∂λ/∂T, but did not cause a change in slope. Using these results, a compact thermo-optic model for predicting thermal rollover was presented, and the laser thermal resistance was calculated ($R_{\mathrm{th}}=$75.9 ± 16.3 K/W). With the thermal resistance, a finite element model of the laser was validated. Using this model, sensitivity analysis of the laser temperature was performed with respect to the boundary conditions. From the modelling, we concluded the following:

1. The flip-chip bonding after BOX layer removal formed a good thermal contact between laser and SiPho, as most of the temperature gradient was situated inside the laser itself.
2. Adding a heat sink created an additional heat transfer path through the top side of the laser and could decrease laser temperature by up to −39%.
3. Adding an underfill below the laser created a thermal shunt for heat flow into the Si substrate, lowering the laser temperature by up to −31%.
4. Thermal coupling to the SiPho die was substantial (∼10%) and could not be ignored; however, this could be cut down in half with the addition of a heat sink.

## Figures and Tables

**Table 1:** Fitting parameters for a compact laser model extracted from experimental results.

Fitting Parameter	$T_{0}$	$I_{0}$	$T_{1}$	$\eta_{0}$
	58	9.6	83	0.18
	(°C)	(mA)	(°C)	(W/A)

**Table 2:** Overview of material properties used for simulation. Cu/Ni/Sn was used for the solder bump metallisation, and Au p- and n-contacts on the laser.

Thermal Conductivity (W/(m·K))	Si	SiO${}_{2}$	Cu/Ni/Sn	Cu	Au	SiN	InP
	150	1	50	400	100	43	68

## Data Availability

The authors can share data upon reasonable request.

## Abbreviations

The following abbreviations are used in this manuscript:

DFB	Distributed feedback
SiPho	Silicon photonics
I/O	Input and output
WDM	Wavelength division multiplexing
BOX	Buried oxide
LIV	Light–current–voltage
MQW	Multiquantum well
WPE	Wall plug efficiency
SOI	Silicon on insulator
